# An *in vitro* model of foam cell formation induced by a stretchable microfluidic device

**DOI:** 10.1038/s41598-019-43902-3

**Published:** 2019-05-16

**Authors:** Xiaoyang Gu, Shijie Xie, Dandan Hong, Yongsheng Ding

**Affiliations:** College of Life Sciences, University of Chinese Academy of Sciences, 19A Yuquan Road, Beijing, 100049 China

**Keywords:** Lab-on-a-chip, Atherosclerosis

## Abstract

Although a variety of animal models of atherosclerosis have been developed, these models are time-consuming and costly. Here, we describe an *in vitro* model to induce foam cell formation in the early stage of atherosclerosis. This model is based on a three-dimension co-culture system in a stretchable microfluidic device. An elastic membrane embedded in the microfluidic device is capable of delivering nonuniform strain to vascular smooth muscle cells, endothelial cells and monocytes adhering thereto, which are intended to mimic the biological environment of blood vessels. Under low-density lipoprotein and stretch treatment, foam cell formation was successfully induced in co-culture with changes in mRNA and protein expression of some related key factors. Subsequently, the model was used to assess the inhibitory effect of atorvastatin on foam cell formation. The results obtained indicate that atorvastatin has a significantly dose-dependent inhibition of foam cell formation, which can be explained by the changes in mRNA and protein expression of the related factors. In principle, the model can be used to study the role of different types of cells in the formation of foam cells, as well as the evaluation of anti-atherosclerotic drugs.

## Introduction

Atherosclerosis, a chronic cardiovascular disease, is mainly caused by hyperlipidemia, hypertension, and diabetes^1,2^. Foam cell formation characterized by accumulation of lipids in cells is a hallmark of early stage of atherosclerosis. Several high-risk factors, such as hypercholesterolemia, hypertension, smoking, and diabetes, can cause endothelial cell dysfunction or damage^3^. Damaged endothelial cells release the inflammatory cytokines to recruit monocytes which penetrate through endothelium and then differentiate into macrophages. When the differentiated macrophages ingest oxidized low-density lipoprotein (ox-LDL) through multiple scavenger receptors LOX-1, CD36, and SR-A1, and cholesteryl ester in lysosomes is hydrolyzed by lysosomal acid lipase (LAL). On the other hand, free cholesterol is esterified with acyl-CoA cholesterol acyltransferase 1 (ACAT1) and vice versa cholesteryl ester is hydrolyzed by neutral cholesteryl ester hydrolase (nCEH) and cholesteryl ester hydrolase (CEH)^4,5^. Since excessive free cholesterol is toxic to cells, it needs to be efficiently removed out of cell via ABCA1 and ABCG1 transporters^6,7^. If the above-mentioned cholesterol homeostasis is disturbed, excessive cholesterol ester or free cholesterol will be accumulated, thereby causing foam cell formation or cell necrosis.

Through administering high-fat diet and knocking out apolipoprotein E gene, a variety of animal models of atherosclerosis have been developed in the past decades, such as rabbit^8^, mice^9^, rat^10^, and pig^11^ models. Although these models help to reveal the mechanisms of atherosclerosis and discover new drug, they are time-consuming and expensive. Therefore, it is still a continuing demand to develop an *in vitro* model of foam cell formation with high efficiency and low cost. From a physiological point of view, vascular smooth muscle cells (VSMCs), endothelial cells (ECs) and monocytes are three types of the main cells involved in atherosclerosis; therefore, it is preferable to establish a co-culture system including these cells for studying foam cell formation. So far, some co-culture systems containing VSMCs and ECs were reported^12–14^, but few of them were applied for the study of foam cell formation.

Under physiological conditions, ECs are mainly affected by two types of mechanical forces, namely shear stress and circumferential stretch. Specially, shear stress generated by blood flow is anti-atherogenic, while circumferential stretch caused by hydrostatic pressure is pro-atherogenic. As one of the major risk factors for atherosclerosis, hypertension not only causes excessive circumferential stretch to arterial wall^15,16^, but also increases production of reactive oxygen species (ROS)^17–20^. Up to now, many clinical evidences suggest that atherosclerosis is prone to occur in arterial bending and branching sites, where shear stress and stretch are characterized with disturbance and unevenness, respectively^21^. Although several studies on the effect of shear stress on vascular endothelial cells have been reported^21–25^, few of them are able to simulate disturbed shear stress and uneven circumferential stretch of atherosclerosis-prone site. Lately, our group introduced a stretchable microfluidic device with the ability to provide non-uniform stretch and somewhat disturbed shear stress to cells^26^.

As an inhibitor of cholesterol biosynthesis, atorvastatin is one of the most widely used drugs to prevent atherosclerosis^27–30^. The main efficacy of atorvastatin includes decreasing blood lipid level, stabilizing plaque^31^, and reducing intracellular ROS^32–34^. However, its effect on foam cell formation has not been reported. In this study, we not only used a stretchable microfluidic device to induce foam cell formation under LDL and stretching treatment, but also applied it for evaluating the efficacy of atorvastatin to inhibit foam cell formation.

## Results

### Constructing the co-culture system

**Figure 1:**
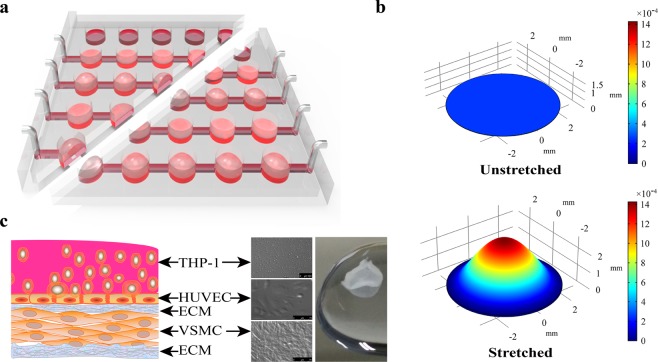
Description of the stretchable microfluidic device and co-culture model. (**a**) The device was fabricated with a sandwich-like structure containing three polydimethylsiloxane layers. Before stretching, the channels and chambers were filled with PBS, while one side of the outlets was blocked and another side of the outlets was connected to a syringe pump. During stretching, when a certain volume of PBS was injected into the chambers, the flat bottom of a well became a spherical cap, resulting in the deformation of the film on which the cells were cultured. Cyclic stretching was accomplished by the alternation of the injection and suction of PBS into and out of chamber, respectively. (**b**) The fitting deformation characteristics of the middle layer (film) in a well of the device during the stretched and unstretched status. During stretched, the film provides an axisymmetric and nonuniform strain to the cultured cells. During unstretched, the film has no strain to the cultured cells. (**c**) Diagram of the co-culture model, microscopic morphology of three types of cells, and a sample of the synthetic multi-type cell membrane. The model was constructed layer by layer under the treatment of 50 μg/mL ascorbic acid to promote the secretion of extracellular matrix by VSMCs.

As reported previously^26^, the microfluidic device was made of three layers of polydimethylsiloxane (PDMS) and cells were seeded on the middle layer which was a thin film to provide an axisymmetric and nonuniform strain to the cells cultured on it, as shown in Fig. 1a,b. Referring to the structural feature of vascular wall which is composed of the intima (endothelium), the media (smooth muscle layer) and the adventitia, a co-culture system consisting of VSMCs, ECs and monocytes was constructed in the experiment (Fig. 1c). First, an integrated microfluidic device was completely sterilized and each well was coated with 20 μg/mL fibronectin (Corning, USA). Second, each well incubated 8.0 × 10^3^ T/G-HA-VSMC cells in the culture medium containing 50 μg/mL vitamin C (Sigma-Aldrich) for 24 h to form a VSMC layer. Third, 1.0 × 10^4^ PUMC-HUVEC-T1 cells were added and incubated for 24 h to form a VSMC + EC co-culture layer and 1.0 × 10^4^ THP-1 cells were added into the co-culture medium before stretching.

### Identifying the co-culture of VSMC + EC

**Figure 2:**
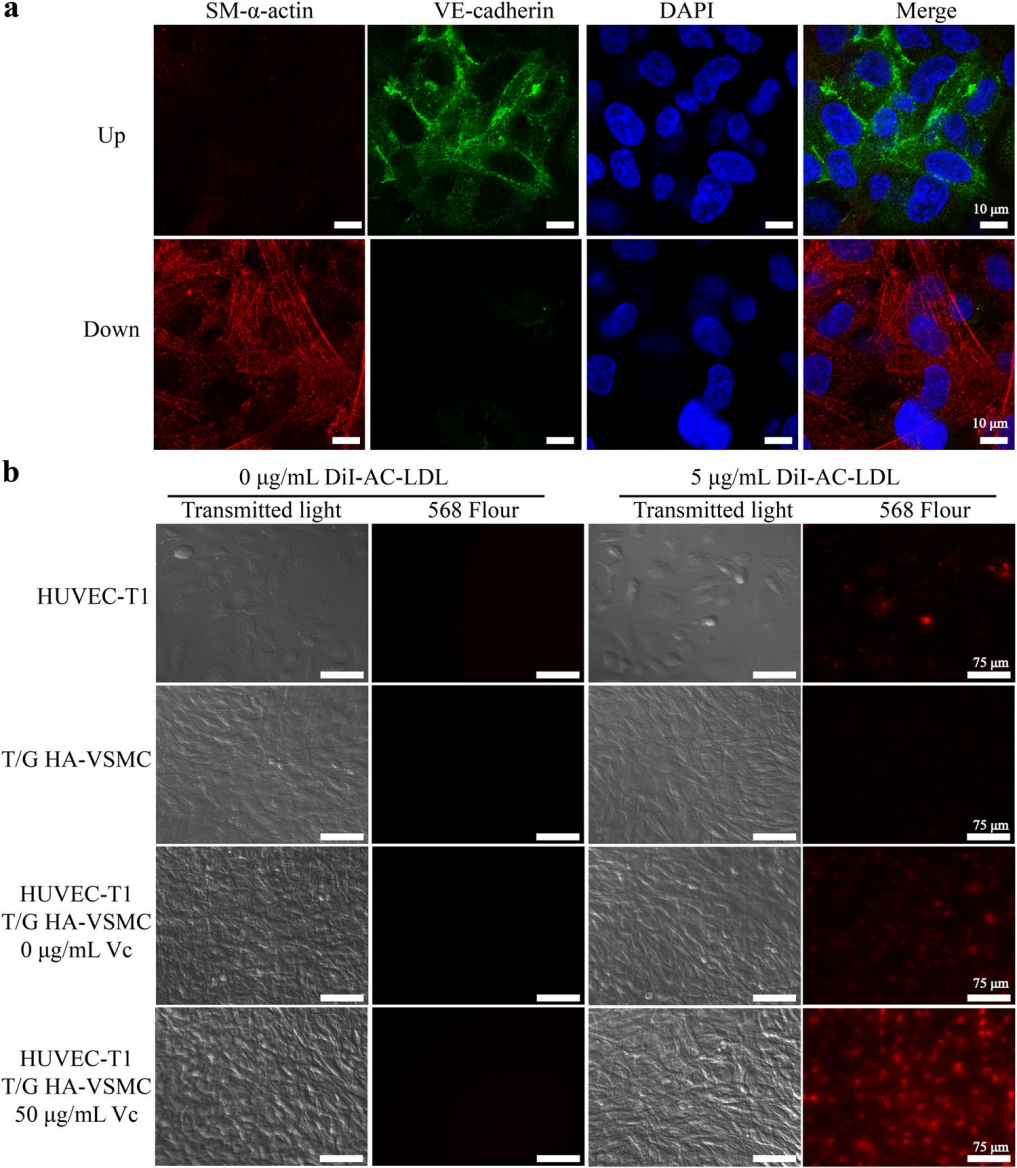
Identification of the co-culture of VSMC + EC. (**a**) Identification of the co-culture model by laser confocal microscopy. The antibodies of VE-cadherin and SM-α-actin were used to identify ECs and VSMCs in the immunofluorescence experiment, respectively. (**b**) Identification of the co-culture model using EC-specific uptake of Ac-LDL function. The uptake of red fluorescence-labeled DiI-Ac-LDL was used to evaluate the EC layer. After establishment of VSMC + EC co-culture, the co-culture system with THP-1 cells was treated under different conditions for 48 h.

The co-culture system of VSMC and EC can be confirmed in terms of structure and function. Structurally, the co-cultured VSMCs and ECs were identified by their specific fluorescence-labeled antibodies of VE-cadherin and SM-α-actin, respectively. As shown in Fig. 2a, the confocal images displayed the red-labeled SM-α-actin of VSMCs and the green-labeled VE-cadherin of ECs at the different focal planes. A series of consecutive images in Supplementary Fig. S1 further reflected the layered structure of the co-culture. Functionally, the uptake of DiI-Ac-LDL can be used to confirm the EC layer because the active uptake of DiI-Ac-LDL is a well-known characteristic of EC^35^. As shown in Fig. 2b, it was apparent that DiI-Ac-LDL was absorbed by the EC monoculture rather than the SMC monoculture. In VSMC + EC co-cultures, the amount of DiI-Ac-LDL accumulated was significantly increased, and it was more obvious under vitamin C treatment condition.

### Inducing the foam cell formation

**Figure 3:**
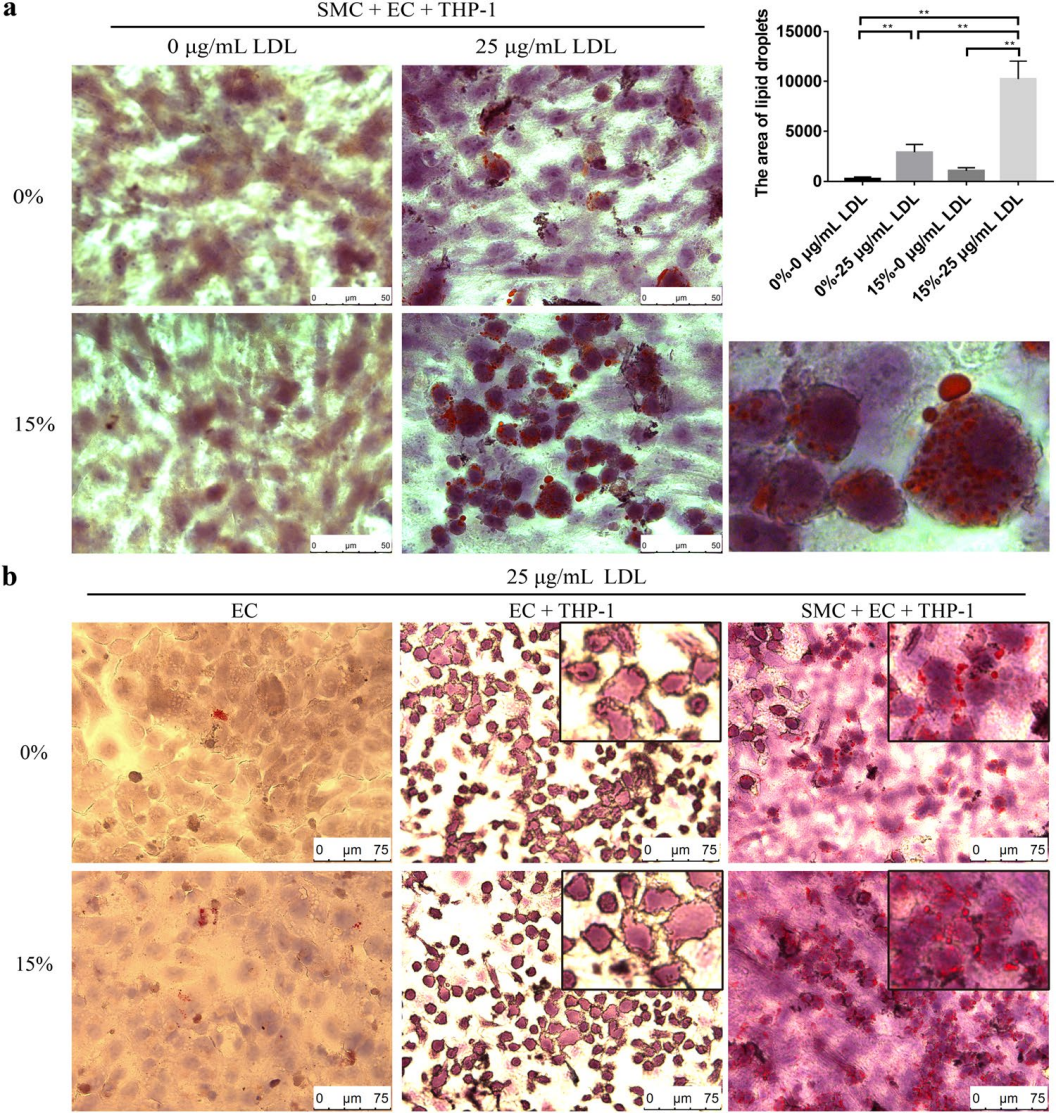
Foam cell formation under LDL and stretch conditions. (**a**) Foam cell formation under four orthogonal experimental conditions. After establishment of VSMC + EC co-culture, the co-culture system with THP-1 cells was treated under different conditions for 48 h, followed by Oil Red O staining. The images were obtained by Leica microscope at × 63 bright field. The tool color range of Photoshop software was used to count lipid droplets. (**b**) The results of foam cells formation under different types of culture/co-culture models with Oil Red O staining. The images were obtained by a Leica microscope at × 40 bright field. The data were shown as mean ± SD (*p < 0.05, **p < 0.01).

**Figure 4:**
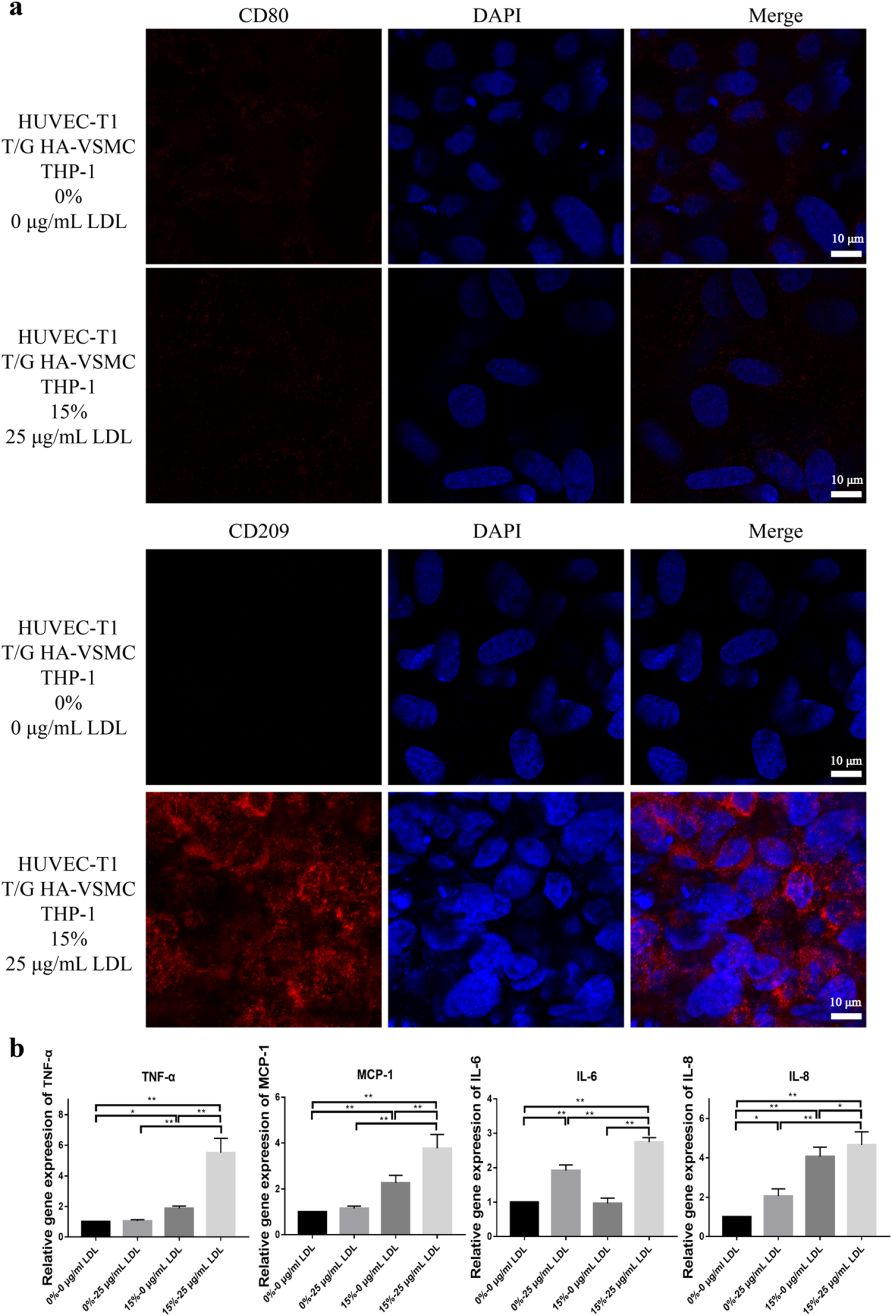
Identification of monocyte differentiation. (**a**) Identification of M1 macrophage by CD80 antibody and dendritic cell by CD209 antibody. (**b**) Relative gene expression of the co-culture model under the different conditions. Quantitative real-time PCR assays of TNF-α, MCP-1, IL-6, IL8 and 18S were performed and the expressions of the target genes were analyzed through ΔCT method. The data were shown as mean ± SD (*p < 0.05, **p < 0.01).

In this experiment, the foam cell formation was induced by the coordination of LDL and stretch. As shown in Fig. 3a, the foam cell formation with 25 μg/mL LDL and 15% stretch together was more pronounced than that with 25 μg/mL LDL alone or 15% stretch alone. In addition, the effect of more different LDL concentrations and different degrees of deformation on foam cell formation can be seen in Supplementary Fig. S3. Figure 3b shows the effect of VSMCs on the foam cell formation in the co-culture. There were only a few Oil Red O stained cells in the EC monoculture and EC + THP-1 co-culture, whereas a large number of Oil Red O stained cells appeared in the EC + VSMC + THP-1 co-culture. From the appearance of the cells in Fig. 3b, most of the cells containing Oil Red O stained droplets were derived from monocytes, and the contours of these cells became irregular. Figure 4a is the identification of the differentiated monocytes by using CD80 (M1 macrophage) and CD209 (M2 macrophage and dendritic cell) antibodies. The results showed that CD209 was significantly expressed in the foam cell model, while CD80 was not detected. In addition, compared with the negative control, the mRNA expressions of the inflammatory factors (TNF-α, MCP-1, IL-6 and IL-8) were increased in three different conditions of LDL, stretch, and LDL + stretch, as shown in Fig. 4b.

### Investigating the mechanism of foam cell formation

**Figure 5:**
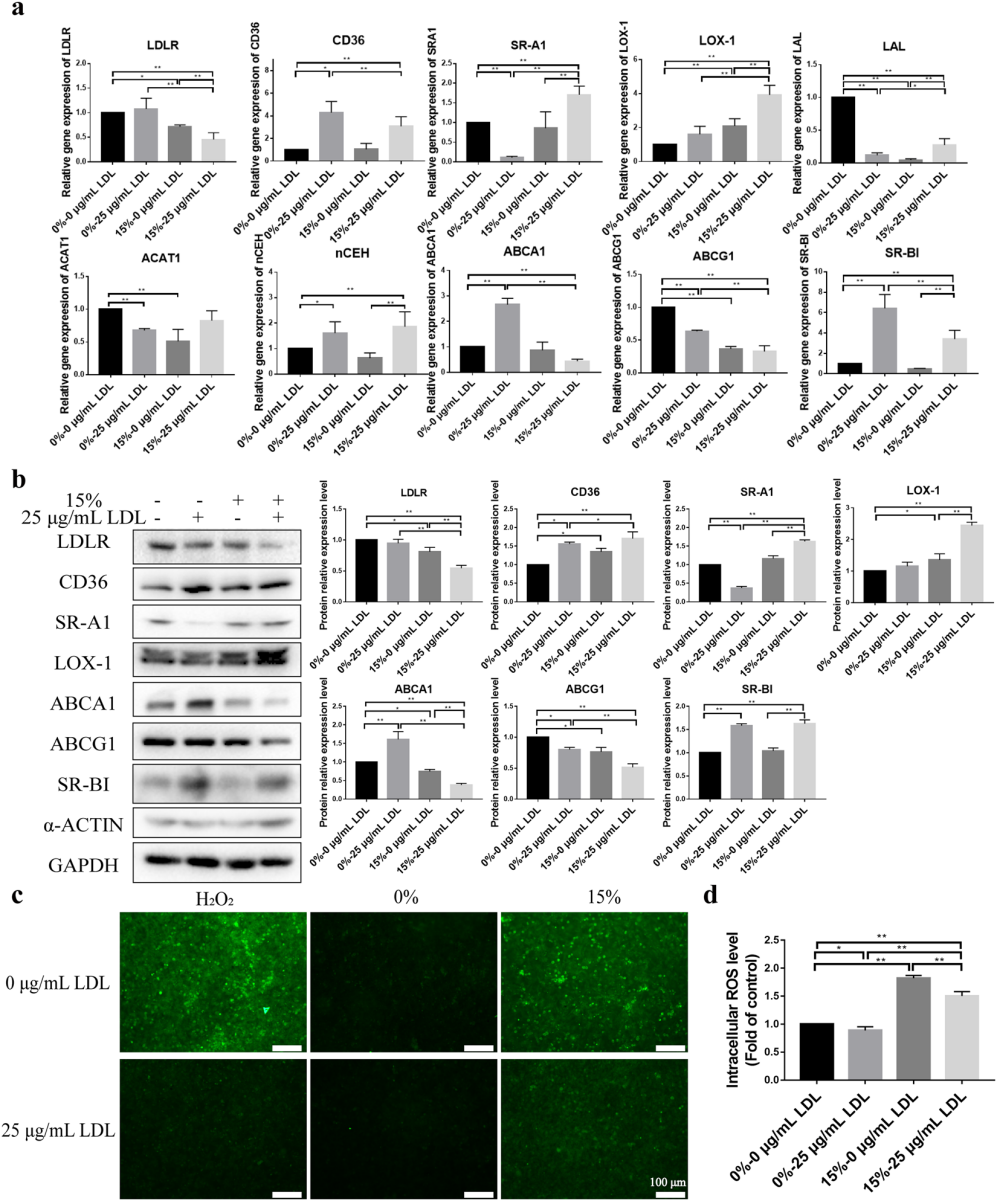
Roles of stretch and LDL in foam cell formation. (**a**) Relative gene expression of the factors associated with foam cell formation under LDL and stretch conditions. Quantitative real-time PCR assays of LDLR, CD36, SR-A1, LOX-1, LAL, ACAT1, nCEH, ABCA1, ABCG1, SR-BI and 18S were performed with extracting the total RNA in the co-cultures. The expressions of the target genes were analyzed through ΔCT method. (**b**) Protein expression of the factors associated with foam cell formation under LDL and stretch conditions. Western blot assays of LDLR, CD36, SR-A1, LOX-1, ABCA1, ABCG1, SR-BI, α-ACTIN and GPADH were performed with extracting the total intracellular protein in the co-cultures. (**c**) Intracellular ROS level measured by a fluorescence microscopy. (**d**) Intracellular ROS level measured by a fluorescence multi-well plate reader. The data were shown as mean ± SD (*p < 0.05, **p < 0.01).

Using qPCR and western blot, the mRNA and protein expression of a series of the related factors, including LDL receptor (LDLR), ox-LDL scavenger receptors (CD36, SR-A1 and LOX-1), cholesterol acyltransferase (ACAT1) and cholesteryl ester hydrolases (nCEH and LAL), and free cholesterol efflux transporters (ABCA1, ABCG1 and SR-BI), were examined under 15% stretch, 25 μg/mL LDL, and the combination of two in comparison with the negative controls. As shown in Fig. 5a,b, the 25 μg/mL LDL alone significantly up-regulated both mRNA and protein expression of CD36, ABCA1 and SR-BI, while it significantly down-regulated both mRNA and protein expression of SR-A1 and ABCG1. The 15% stretch alone significantly down-regulated both mRNA and protein expression of LDLR and ABCG1, while it significantly up-regulated both mRNA and protein expression of LOX-1. The combination of LDL and stretch significantly up-regulated both mRNA and protein expression of CD36, SR-A1, SR-BI and LOX-1, while it significantly down-regulated both mRNA and protein expression of LDLR, ABCA1 and ABCG1. Additionally, the changes in the intracellular ROS level under the different conditions were observed in Fig. 5c,d. The stretch promoted the increase of intracellular ROS levels. Under the same other conditions, LDL-treated intracellular ROS levels were lower than those without LDL treatment.

### Inhibiting the foam cell formation by atorvastatin

**Figure 6:**
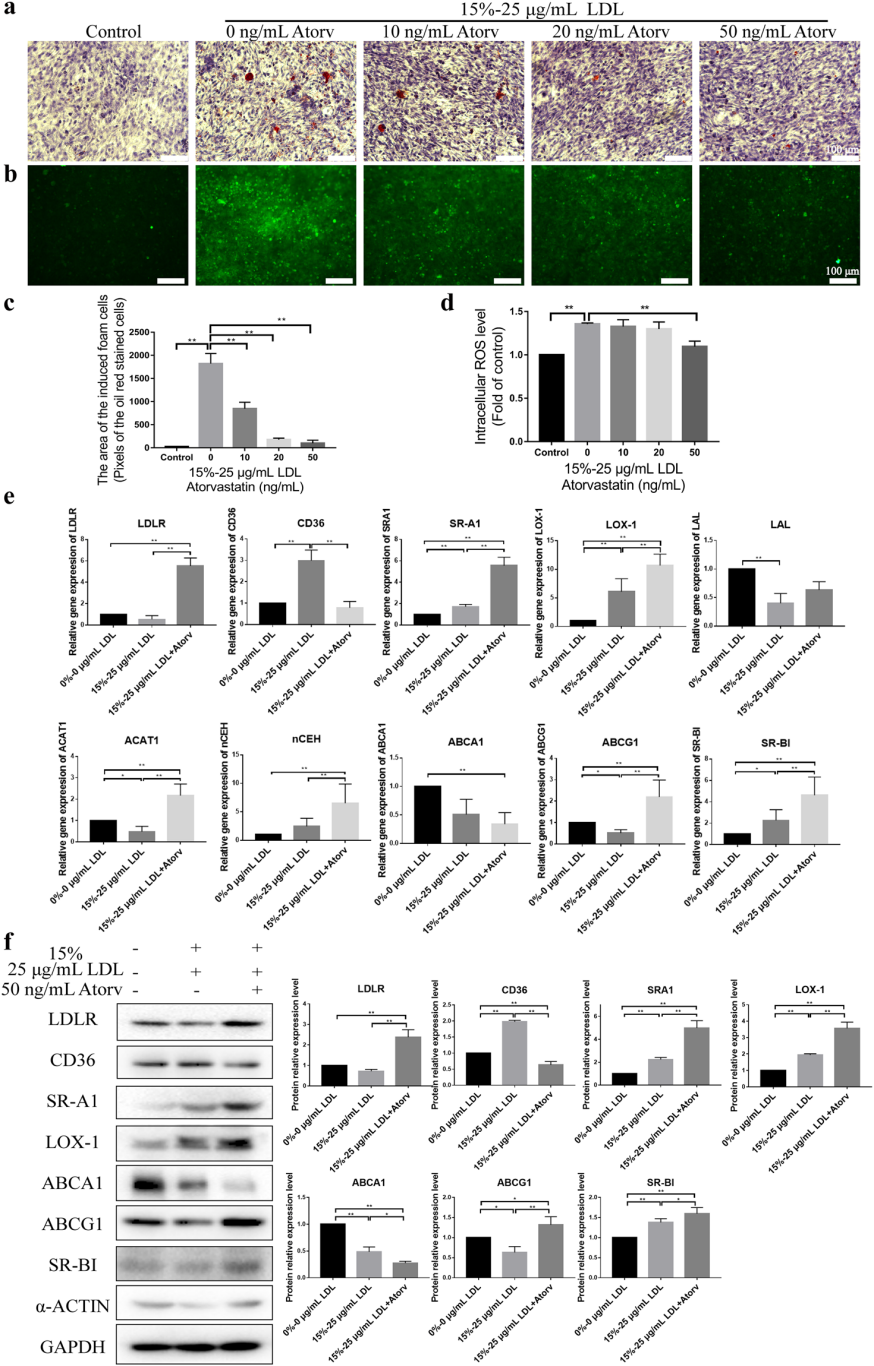
Inhibition of foam cell formation by atorvastatin. (**a**) Effect of different concentration of atorvastatin on foam cell formation. Oil Red O staining was performed to identify the foam cell formation under the different concentration of the drug. The images were obtained by a Leica microscope at × 20 bright field. (**b**) Effect of different concentration of atorvastatin on ROS level. (**c**) Statistical analysis of the lipid droplets generation for (**a**) experiment. (**d**) Statistical analysis of intracellular ROS level for (**b**) experiment. The total green fluorescence intensity of each well was quantified by using a fluorescence multi-well plate reader. (**e**) The mRNA expression of the related factors in the co-culture model under different conditions. The method was the same as in Fig. 5a. (**f**) The protein expression of the related factors in the co-culture model under different conditions. The method was the same as in Fig. 5b. The data were shown as mean ± SD (*p < 0.05, **p < 0.01).

To explore the usefulness of this model, atorvastatin was selected to evaluate its effect on the formation of foam cells. As shown in Fig. 6a–d, atorvastatin exhibited a significantly dose-dependent efficacy on the inhibition of foam cell formation and the attenuation of ROS production. Figure 6e,f showed that atorvastatin significantly increased both mRNA and protein expression of LDLR SR-A1, LOX-1, SR-BI and ABCG1, while it significantly decreased mRNA and protein expression of CD36 and ABCA1. In addition, atorvastatin significantly up-regulated the mRNA expression of ACAT1 and nCEH, but the up-regulation of nCEH mRNA expression was much greater than that of ACAT1 mRNA expression.

## Discussion

A detailed description of the non-uniform tensile deformation provided by the device can be found in our previously reported literature^26^. To the best of our knowledge, this is the first time the device has been used to establish a foam cell formation model under multi-type cell co-culture conditions. Figure 1a shows the structure of the chip which is divided into an upper well and a lower chamber separated by a layer of elastic film. When a certain amount of liquid is injected into the lower chamber, the film bulges upward to form a spherical cap which produces a central axisymmetric nonuniform deformation distribution, as shown in Fig. 1b. The deformation with this characteristic is somewhat similar to the case of a blood vessel bending site prone to atherosclerosis. In addition, it should be noted that the perturbation of the medium in the well caused by the cyclic deformation of the elastic membrane can also simulate the intravascular blood flow disturbance to some extent. Figure 1c shows a schematic representation of a multicellular co-culture structure and a real sample membrane. The above features are intended to mimic the actual physiological environment of blood vessels to establish the foam cell formation model.

The laser confocal images in Fig. 2a indicate that the green fluorescently labeled EC-specific VE-cadherin is located in the upper layer of the thin film tissue, while the red fluorescently labeled SMC-specific SM-α-actin is located in the lower layer of the thin film tissue. This result demonstrates that the white film (shown in Fig. 1c) is a layered structure composed of VSMC and EC cells similar to the inner wall of the blood vessel. Acetylated LDL (Ac-LDL) acts as a substance that is specifically taken up by EC. We further characterized the VSMC and EC composite membranes formed by co-culture using fluorescently labeled DiI-Ac-LDL. As shown in Fig. 2b, the red fluorescently labeled DiI-Ac-LDL was taken up by EC monoculture and EC + VSMC co-cultures rather than VSMC monoculture, and the amount of DiI-Ac-LDL absorbed by vitamin C-treated SMC + EC co-culture was the highest. This result is due to the fact that vitamin C promotes the secretion of extracellular matrices by smooth muscle cells, in which proteoglycans help to retain DiI-Ac-LDL for EC uptake. It indicates that LDL retention may facilitate the formation of foam cells in vitamin C-treated SMC + EC co-culture.

Since hyperlipidemia and hypertension are two important factors leading to atherosclerosis, we introduce LDL and stretching into the co-culture to establish the model. In the experiment, LDL was directly added to the medium to simulate hyperlipidemia and the deformation of the film provided a stretch stress to simulate hypertension. Figure 3a shows the experimental results of foam cell formation under the cross-design conditions of LDL and stretch. There are two points to be pointed out here. First, the formation of foam cells is the most prominent under the combined condition of LDL and stretch. Second, the formation of foam cells is not obvious without LDL application, that is, LDL is more important than stretching. In addition, it should be noted that the native LDL is used in this experiment instead of ox-LDL commonly used in the conventional method of inducing foam cell formation. This may better simulate the process of modifying LDL in the body. Some studies hypothesize that LDL captured in the subendothelial space during plaque formation is oxidized to form ox-LDL. Unlike other methods using only monocyte to induce foam cell formation, the model is based on the co-culture of multiple types of cells involved in foam cell formation *in vivo*. Figure 3b shows the difference in EC, THP-1, and VSMC affecting foam cell formation. In EC monocultures, few cells were stained with Oil Red O. In EC + THP-1 co-culture, although a large number of monocytes adhered to endothelial cells, no oil red O stained droplets were observed in hematoxylin-stained monocytes. However, in EC + THP-1 + VSMC co-culture, significant Oil Red O stained droplets appeared in a large number of adherent monocytes. It can be concluded that VSMCs play an important role in this *in vitro* foam cell formation model through cytokines and extracellular matrices. It has been generally accepted that monocytes differentiate into macrophages and then evolve into foam cells. In this model, CD80 was not detected, but CD209 was observed (Fig. 4a). Although it has also been reported that CD209 recognizes M2 macrophages, here we prefer to identify dendritic cells because of the significant increase in the expression of some major inflammatory factors, such as TNF-α, MCP-1, IL-6 and IL-8 (Fig. 4b).

Undoubtedly, for foam cell formation, LDL is an internal cause and stretching is an external cause. This rule can also be seen from the results of the expression changes of these relevant factors in Fig. 5, such as lipoprotein receptor (LDLR), scavenger receptors (CD36, SR-A1, and LOX-1), cholesterol efflux transporters (ABCG1 and ABCA1), and cholesterol invertases (ACAT1, nCEH, and LAL). As shown in Fig. 5a,b, the significant evidences closely related to foam cell formation were the up-regulation of CD36, LOX-1, and SR-A1 and the down-regulation of ABCG1 and ABCA1. The synergistic effect of LDL and stretching promoted foam cell formation as the expression of LOX-1 and ABCG1 were significantly up-regulated and down-regulated in the same direction, respectively. As an anti-atherosclerotic factor, LDLR showed a downward trend under the conditions that promoted foam cell formation. Despite some contradictory results, the idea that ox-LDL leads to foam cell formation is still prevalent. Figure 5c shows that stretch can increase intracellular ROS levels, whereas LDL is just the opposite. Therefore, the estimation of the increase in ROS caused by stretching to promote the oxidation of LDL and the formation of foam cells is consistent with the above viewpoint. On the other hand, the expressions of the ox-LDL-specific receptors SR-A1 and LOX-1 were significantly up-regulated under the combined conditions of stretching and LDL, which may also be a response to the accumulation of ox-LDL. The mRNA expressions levels of cholesterol esterase (ACAT1) and cholesterol ester hydrolase (LAL) were generally inhibited under stretch and LDL conditions, except for nCEH. In general, the interconversion between cholesterol esterification and cholesterol ester hydrolysis determines that intracellular cholesterol is retained in ester form or is eliminated in free form.

Atorvastatin was chosen to demonstrate the potential application of this foam cell formation model for anti-atherosclerotic drug discovery. As shown in Fig. 6a–d, atorvastatin showed a dose-dependent inhibition on the foam cell formation and a decrease in the intracellular ROS level. The data in Fig. 6e,f showed that atorvastatin significantly down-regulated CD36 and ABCA1 expression and up-regulated LDLR, ABCG1, SR-A1, LOX-1, SR-BI expression, as well as mRNA expression of ACAT1 and nCEH. It is worth noting that the expression trends of LDLR, CD36, ABCG1 and ACAT1 under the administration and non-administration were opposite in comparison with the negative controls. These effects of inhibiting the formation of foam cells can be explained as follows: up-regulation of LDLR promotes increased uptake of protective LDL, down-regulation of CD36 leads to decreased intake of modified LDL, up-regulation of ABCG1 directly promotes efflux of free cholesterol, and up-regulation of ACAT1 increases cholesterol metabolic activity. Figure 6b showed that increasing the drug concentration reduced intracellular ROS level. This may help reduce the production of ox-LDL. It is also important to note that the down-regulation of CD36 expression and the up-regulation of other scavenger receptors (LOX-1 and SR-A1) under administration conditions are contradictory to the interpretation of foam cell formation, indicating the complexity of the mechanism. Nevertheless, this *in vitro* foam cell formation model can be used to confirm the inhibitory effect of atorvastatin on foam cell formation.

**Figure 7:**
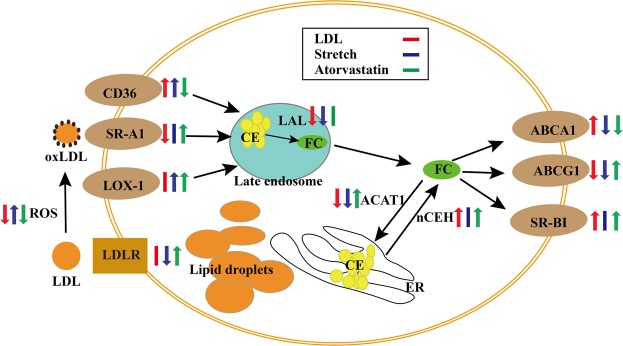
Summary of effects of LDL, stretching and atorvastatin on foam cell formation. Abbreviation: LDL, low-density lipoprotein; ox-LDL, oxidized low-density lipoprotein; ROS, reactive oxygen species; CE, cholesterol ester; FC, free cholesterol; LOX-1, oxidized low-density lipoprotein receptor 1; CD36, cluster of differentiation 36, also known as platelet glycoprotein 4, fatty acid translocase (FAT), and scavenger receptor class B member 3 (SCARB3); SR-A1, scavenger receptors type 1; LAL, lysosomal acid lipase, also known as lipase A (LIPA); ACAT1, acyl-CoA: cholesterol acyltransferase 1, also known as sterol O-acyltransferase 1 (SOAT1); nCEH, neutral cholesteryl ester hydrolase; ABCA1, ATP-binding cassette transporter member 1; ABCG1, ATP-binding cassette sub-family G member 1; SR-BI, scavenger receptor class B type 1.

The abovementioned effects of LDL, stretch, and atorvastatin on the foam cell formation are summarized in Fig. 7. Unlike most *in vitro* models using monocytes, chemical inducers, and ox-LDL to induce the form cells under the static culture condition, our model is based on VSMC + EC + THP-1 co-culture and non-uniform strain to mimic the complex physiological environment of vessel, and also can provide a platform to study the role of EC, VSMC, and monocyte on foam cell formation as well as to evaluate anti-atherosclerosis drugs. However, this model only addressed the effects of stretch and LDL on foam cell formation and did not take into account other factors (vascular geometry, humoral environment, metabolic mechanism) affecting the development of atherosclerosis due to the limitation of this model.

## Materials and Methods

### Stretchable microfluidic device system

The microfluidic device was made of three layers of polydimethylsiloxane (PDMS). Briefly, the templates were made of negative photoresist (SU-8 2035) by referring to the method provided by the manufacturer. The two templates of the top and bottom layers contain a 5 × 5 chamber array pattern, and the bottom template has channels that connect the five chambers into a row. After PDMS on the template of the top layer was cured at 65 °C for 1 h, the PDMS top layer was peeled off and punched into an array of holes according to the chamber pattern. The punched top layer was pressured on an incompletely cured PDMS middle layer (100-μm in thickness). After the top and middle layers were re-cured together, the combined layer was carefully peeled off from the wafer. The top-middle layer and the bottom layer were bound together by plasma. Some metal pipe fittings and silicone rubber tubes were used to connect the outside and microchannels, as shown in Fig. 1a. The entire stretchable microfluidic device system includes the following components: a stretchable microfluidic device, an incubator, and a syringe pump. The chip was fixed in a cell dish and one end of the microchannel was connected to the syringe pump and another end of the microchannel was blocked, as shown in Fig. S2b. The working principle of the retractable microfluidic device is shown in Fig. 1b. The strain of the intermediate film is symmetrically distributed around the axis of the spherical cap, and the intensity decreases from the center to the edge. Cyclic stretching was achieved by alternately injecting and withdrawing liquid. More details can be found in Supplementary Fig. S2.

### Cell lines and cell culture

The human aortic vascular smooth muscle cell line (T/G HA-VSMC), which was obtained from American Type Culture Collection (ATCC, Manassas, VA, USA), was a gift by Dr. Chen Jian’s Lab. The human umbilical vein endothelial cell line (PUMC-HUVEC-T1) and human monocytic cell line (THP-1) were purchased from National Infrastructure of Cell Line Resource of China (Beijing, China). T/G HA-VSMC and PUMC-HUVEC-T1 cell lines were maintained in high-glucose DMEM (Life Technologies, Gibco) supplemented with 10% fetal bovine serum (FBS, Life Technologies, Gibco), MEM Non-essential amino acids (MEM NEAA 100×, Life Technologies, Gibco), penicillin (100 U mL^−1^, Gibco, USA) and streptomycin (100 μg mL^−1^, Gibco, USA). THP-1 cells were maintained in RPMI 1640 Medium (Caisson Labs) supplemented with 10% fetal bovine serum (FBS), penicillin (100 U mL^−1^) and streptomycin (100 μg mL^−1^). The cell lines were cultured in cell incubator (37 °C, 5% CO_2_) (Thermo Fisher Scientific). Human DiI-Acetylated LDL and LDL were purchased from Yeasen (Shanghai, China).

### Immunofluorescence assay

The cells were fixed with 4% (v/v) paraformaldehyde (Sigma-Aldrich) in PBS for 15 min and permeabilized with 0.2% (v/v) Triton X-100 in PBS at room temperature. The cells were blocked with 2% (v/v) BSA in PBS and immunostained with primary antibodies (anti-α-Actin, anti-CD80 and anti-CD209 from Santa Cruz Biotechnology and anti-VE-cadherin from Cell Signaling Technology) for 2 h, then followed by the incubation with Alexa Fluor 488- and 568-conjugated secondary antibodies for 2 h and DAPI (0.1 μg/mL) for 3 min. Images were acquired with a confocal microscope (Zeiss, Germany).

### Oil Red O staining

After treated by stretch and LDL (Yeasen, China) for 48 h, the cells were fixed with 4% paraformaldehyde in PBS for 15 min and covered with Oil Red O (Biolabs, China) working solution (0.6% (m/v) Oil Red O in the 60% (v/v) isopropanol solution) for 30 min. The stained cells were incubated with 60% isopropanol for 2 min and followed by a hematoxylin solution (Beijing Biodragon Immunotechnologies, China) for 3 min. Hematoxylin solution was discarded and the cells were washed with tap water for 2–5 times. The sample was covered with PBS and then mounted with a slide. Images were acquired with a microscope (Leica Microsystems). Lipid droplets appeared red and nuclei appeared blue, respectively. The amount of red color was counted by the Photoshop software.

### ROS assay

Dichlorodihydrofluorescein diacetate (DCFH-DA) (Sigma-Aldrich) was used for measuring the intracellular level of reactive oxygen species (ROS). The reagent was dissolved in DMSO to prepare a 10 mM stock solution. An appropriate amount was added to the cell culture system to form a concentration of 10 μM for testing.

After stretched for 24 h, the cells were washed with PBS for three times, followed by incubation of 10 μM DCFH-DA in serum-free medium for 30 min in the cell culture incubator. Then the cells were fixed with 4% paraformaldehyde in PBS for 15 min and washed with PBS for three times. Fluorescence was visualized and photographed by a fluorescent inverted microscope (Leica, DMI 6000B).

After stretched for 24 h, the cells were collected and lysed with ultra-pure water at 4 °C for 30 min. Then the cells were centrifuged at 12,000 rpm for 10 minutes and the supernatant was transferred to a 96-well plate. The total green fluorescence intensity of each well was quantified using a fluorescence multi-well plate reader (Synergy 2 SL) with excitation and emission wavelengths of 485 nm and 525 nm, respectively.

### Real-time PCR analyses

The total RNA in the co-culture was extracted with TRIZOL according to the protocols provided by the manufacturer. The collected RNA was used to generate cDNA by a transcriptor cDNA Synth Kit2 (Roche) with an oligo-dT primer. Quantitative real-time PCR assays of LDLR, CD36, SR-A1, LOX-1, LAL, ACAT1, nCEH, ABCA1, SR-BI, ABCG1, and 18S were performed using an MX3000 real-time PCR instrument (Stratagene) with SYBR Green Mixture (Trans, China). The expressions of target genes were analyzed through ΔCT methods.

### Western blotting assay

Cells lysates were generated by using RIPA buffer (Beyotime, China). The extracted proteins were separated through 8.5% SDS-PAGE gel and transferred onto a PVDF membrane (Millipore). The PVDF was blocked with 5% milk for 1 h. The primary antibodies for LDLR (Abcam, ab52818), CD36 (Proteintech, 18836-1-AP), SR-A1 (Abcam, ab183725), LOX-1 (Proteintech, 11837-1-AP), ABCA1 (Abcam, ab18180), ABCG1 (Abcam, ab52617), SR-BI (Abcam, ab52629), ACTA2 (α-ACTIN) (Santa Cruz, Sc-13606), GAPDH (Abgent, AM1020b) and second antibodies conjugated to HRP were used to recognize the target proteins. The targets were visualized using ECL (Advansta, R-03031-C50) and detected by using an imaging system (Bio-Rad Chemi Doc XR6^+^).

### Statistical analysis

SPSS statistics 25 software was used for analysis and the data were shown as means ± SD of at least five independent experiments. Statistical significance was assessed by one-way analysis of variance (ANOVA) with Duncan’s post-hoc test. Differences were considered significantly with a P-value less than 0.05.

## Supplementary information

Supplementary information
