# Supplementary material for: An in vitro model of foam cell formation induced by a stretchable microfluidic device
Source: Sci Rep. 2019 May 16;9:7461. doi: 10.1038/s41598-019-43902-3 (PMC6522483; doi:10.1038/s41598-019-43902-3)
Supplement: Supplementary file 1 — Supplementary information [file 41598_2019_43902_MOESM1_ESM.pdf]

An in vitro model of foam cell formation induced by a stretchable microfluidic device

Xiaoyang Gu, Shijie Xie, Dandan Hong and Yongsheng Ding*

College of Life Sciences, University of Chinese Academy of Sciences, 19A Yuquan Road, Beijing 100049, China

**Supplement table 1**

List of primer sequences used for real-time quantitative PCR analysis in this study.

| | |
|---|---|
| 18s-Forward | AGTCCCTGCCCTTTGTACACA |
| 18s-Reverse | CGATCCGAGGGCCTCACTA |
| LDLR-Forward | GAGTGAACTGGTGTGAGAGGAC |
| LDLR-Reverse | GTGTGCTGTGTCCTTACGGCTG |
| CD36-Forward | GGACATACTTGGATATTGAACC |
| CD36-Reverse | ACACCAACACTGAGTAAGAT |
| SR-A1-Forward | CCTTTACCTCCTCGTGTTT |
| SR-A1-Reverse | TGTTGCTCATGTGTTCCA |
| LOX-1-Forward | TCTGACCTCCTAACACAAGA |
| LOX-1-Reverse | AGATTCTGGTGGTGAAGTTC |
| LAL-Forward | TAGGACGATTACCAGATCATC |
| LAL-Reverse | AGCAGGAGAATGTGTTGTAT |
| ACAT1-Forward | CGCTGCTGTAGAACCTATT |
| ACAT1-Reverse | CCGTATTCTCCTTGCTTCA |
| NCEH1-Forward | AGCATGATGTCCTCAGAGA |
| NCEH1-Reverse | GCCACTTGATGTAACTATTCC |
| ABCA1-Forward | GGAGATCAGTAGTGCTTACAT |
| ABCA1-Reverse | GCCAGAGAAGATAATGAAGATG |
| ABCG1-Forward | CCTACAGTGGATGTCCTAC |
| ABCG1-Reverse | CCGAGTACGATGAAGTCC |
| SR-BI-Forward | GGTGATGATGGAGAATAAGC |
| SR-BI-Reverse | TAATCCGAACTTGTCCTTGA |
| TNFα-Forward | TCTGGGCAGGTCTACTTTGGG |
| TNFα- Reverse | GAGGTTGAGGGTGTCTGAAGG |
| MCP1-Forward | CCAGCAGCAAGTGTCCCAAAG |
| MCP1-Reverse | TGCTTGTCCAGGTGGTCCATG |
| IL6-Forward | GCCACTCACCTCTTCAGAACG |
| IL6-Reverse | TTTCACCAGGCAAGTCTCCTC |
| IL8-Forward | TTCAGGAATTGAATGGGTTTGC |
| IL8-Reverse | CACTGTGAGGTAAGATGGTGG |

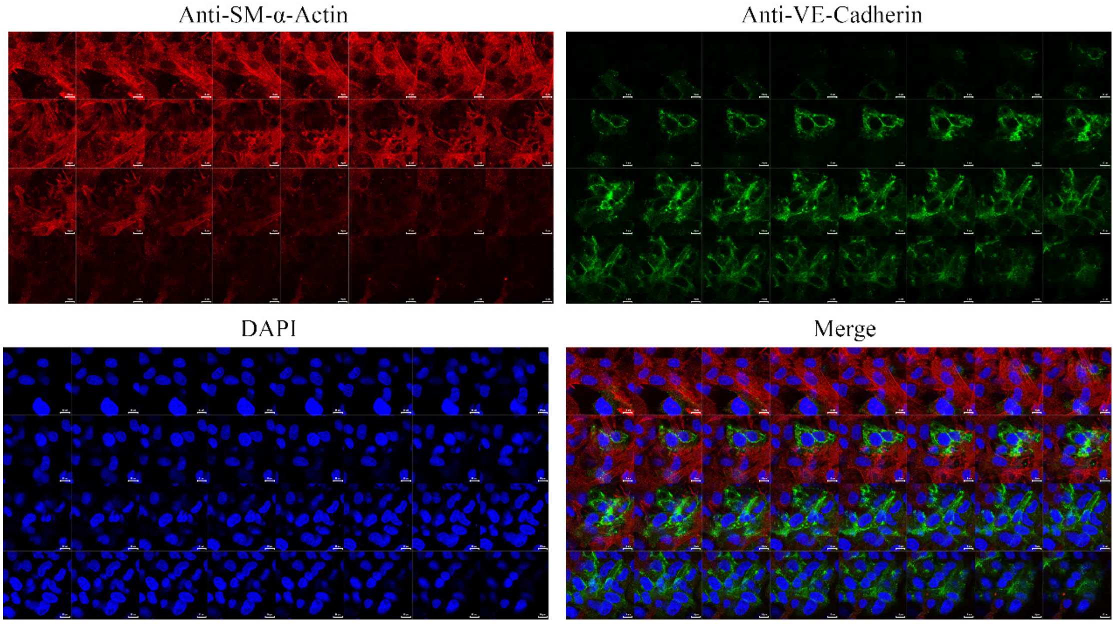


**Supplement Fig. 1.** The entire images of *Z*-axis scanning of the co-culture model. Antibodies of VE-cadherin (protein marker of EC), and SM-α-actin (protein marker of VSMC), were used for immunofluorescence experiments. *Z*-axis scanning of the co-culture model was performed under a laser confocal microscope. With the focus moving up, the VSMCs disappeared while the ECs appeared gradually.

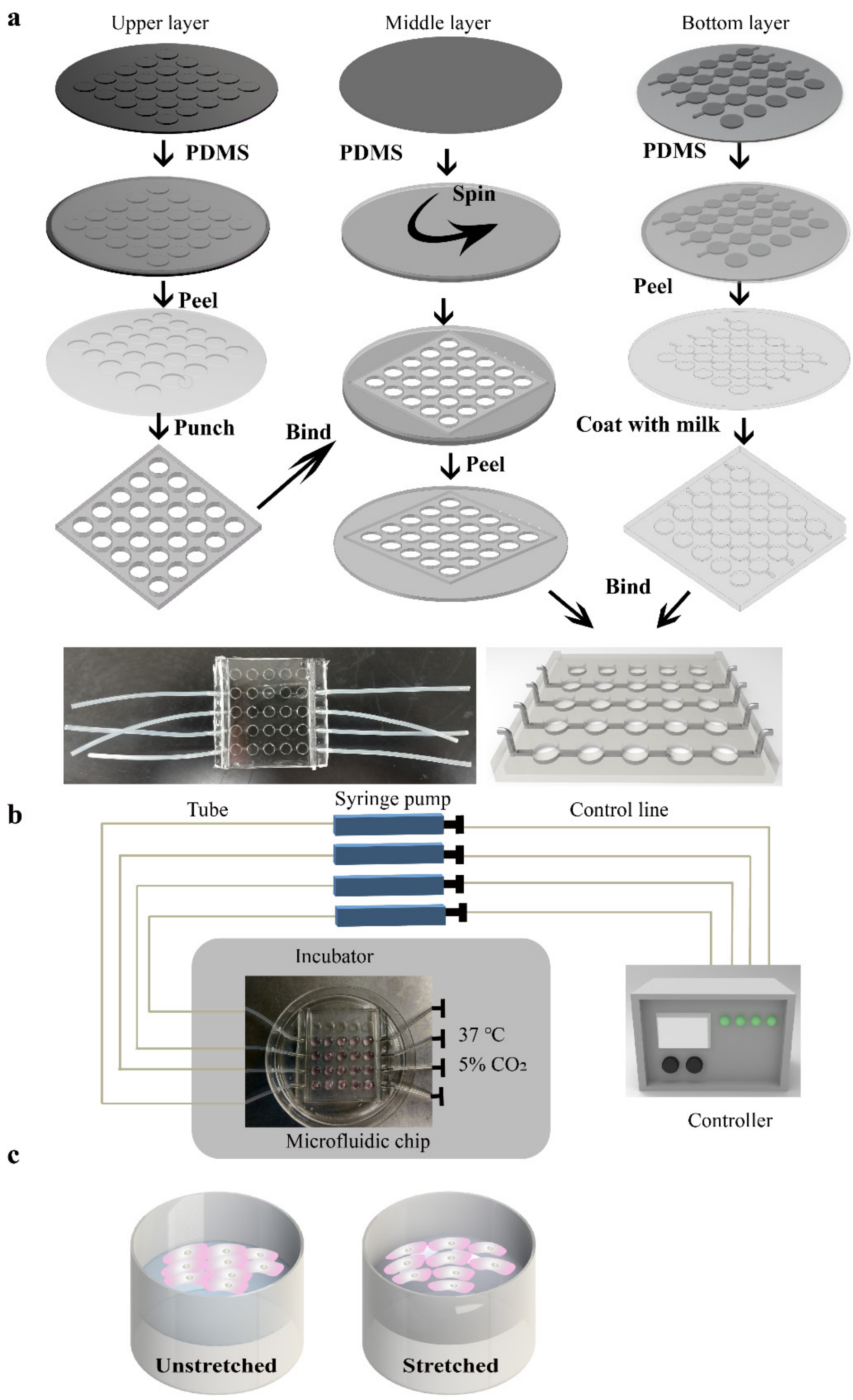


**Supplement Fig. 2.** Description of the stretchable device and its working principle.
(a) Fabrication of the stretchable device and the image of a real device. (b) The scheme of the entire stretching system. The system contains stretchable device, syringe pumps, syringes, tubes and incubator. (c) The working principle of the stretchable device. Two conditions of the stretchable device were showed. When the device was stretched, the middle layer can provide an axisymmetric and nonuniform strain to the cultured cells. When the device was unstretched, the middle layer was flat, there was no strain to the cultured cells.

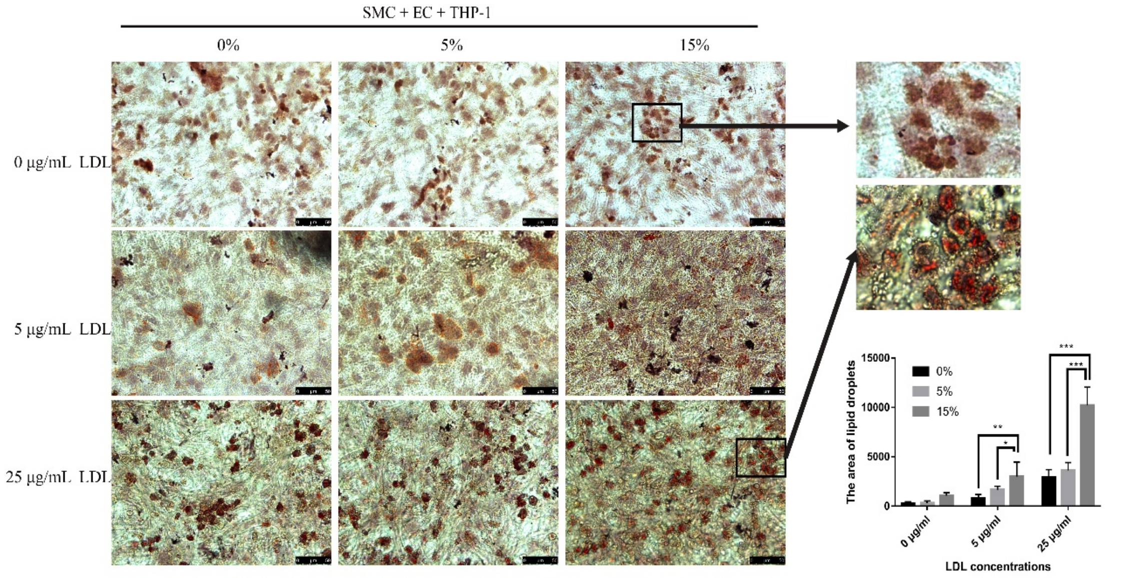


**Supplement Fig. 3.** Foam cells were formed under the treatments of different concentrations of LDL and different degrees of deformation. These treatments lasted for 48 hours since the co-culture model was constructed. Oil-red O stain was performed subsequently. The images were obtained by Leica microscope at ×40 bright field. Statistical analysis of the lipid droplets generation. The tool color range of photoshop software was used to count lipid droplets. Data was shown as mean ± SD. *p < 0.05, **p < 0.01, ***p < 0.001.

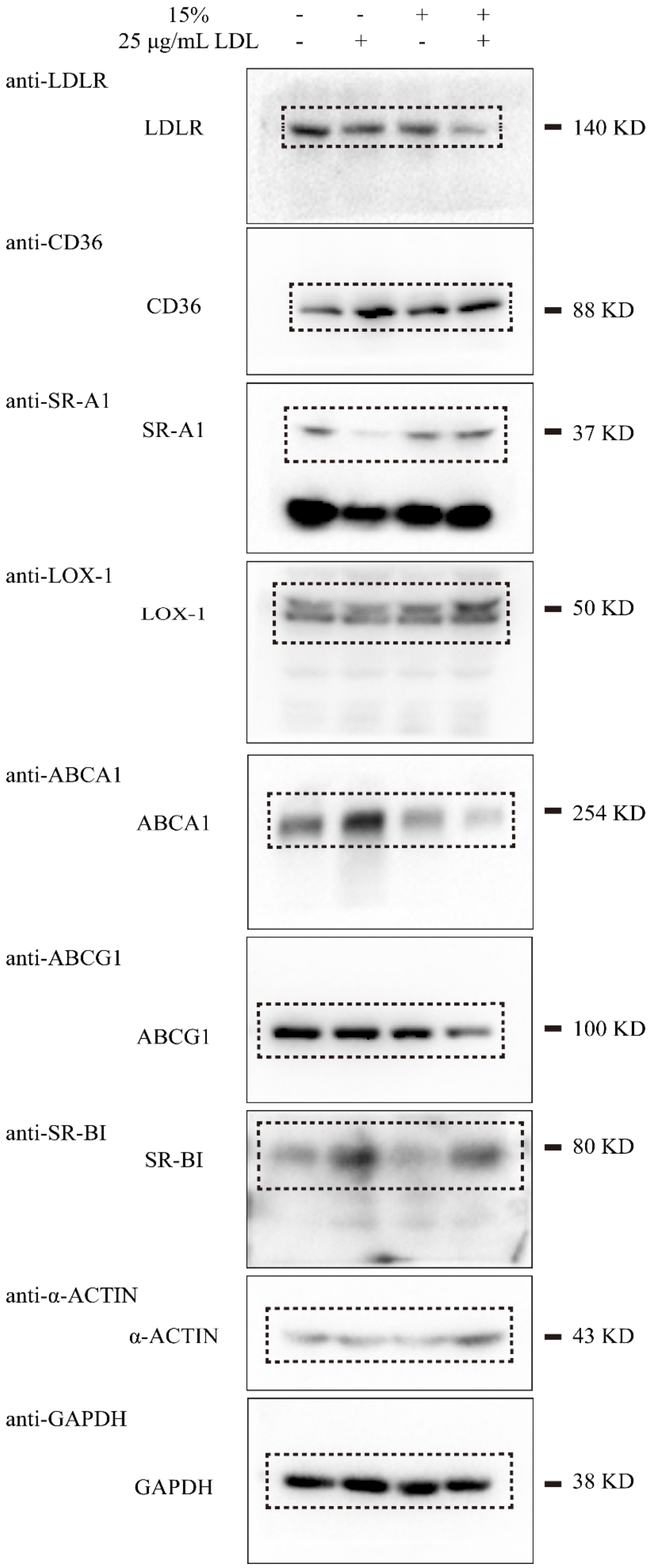


**Supplement FIG.4.** Full-length blots from figure 5b.

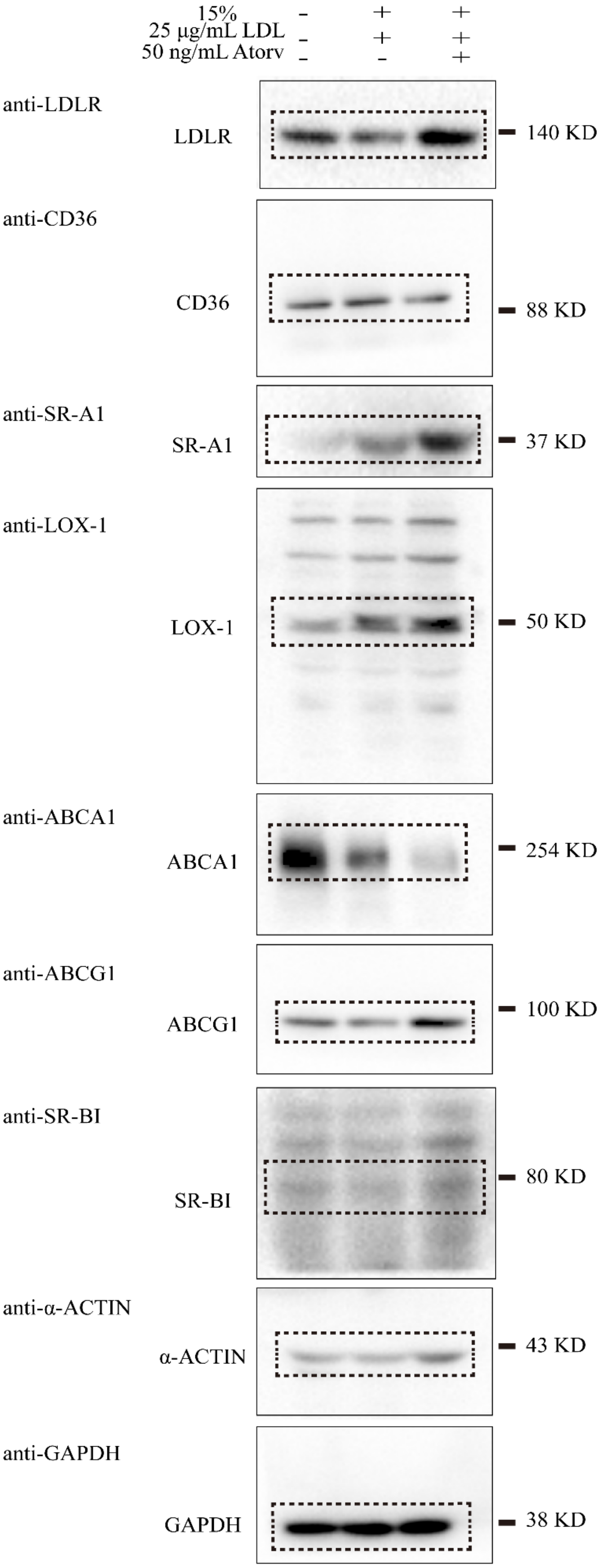


**Supplement FIG.5.** Full-length blots from figure 6f.
